# Oral health status in Sichuan Province: findings from the oral health survey of Sichuan, 2015–2016

**DOI:** 10.1038/ijos.2017.6

**Published:** 2017-03-30

**Authors:** Wei Yin, Ying-Ming Yang, Hong Chen, Xue Li, Zhuo Wang, Li Cheng, Qiu-Dan Yin, Hong-Zhi Fang, Wei Fei, Fang-Lin Mi, Min-Hai Nie, Tao Hu, Xue-Dong Zhou

**Affiliations:** 1 State Key Laboratory of Oral Diseases, National Clinical Research Center for Oral Diseases, Department of Preventive Dentistry, West China Hospital of Stomatology, Sichuan University, Chengdu, China; 2 Sichuan Center for Disease Control and Prevention, Chengdu, China; 3 The Third People's Hospital of Chengdu, Chengdu, China; 4 Sichuan Academy of Medical Sciences & Sichuan Provincial People's Hospital, Chengdu, China; 5 Department of Stomatology, North Sichuan Medical College, First Affiliated Hospital, Nanchong, China; 6 Department of Stomatology, Southwest Medical University, Luzhou, China; 7 State Key Laboratory of Oral Diseases, National Clinical Research Center for Oral Diseases, West China Hospital of Stomatology, Sichuan University, Chengdu, China

**Keywords:** cross-sectional study, dental caries, oral health status, periodontal condition, Sichuan Province of China

## Abstract

To investigate oral health status in the residents of Sichuan Province, southwest China, a cross-sectional study was performed using the latest Oral Health Survey Basic Methods recommended by the World Health Organization. A multistage stratified random cluster-sampling method was used to enroll participants from the following three groups: children aged 3–5 years, adolescents aged 12 years, and people aged 65–74 years. In these three groups, the mean numbers of teeth that were affected by caries were 3.28, 0.86 and 5.13, respectively, resulting in a prevalence of 63.47%, 37.20% and 83.20%, respectively. Relative to the high rate of decayed teeth, the prevalence of fillings was very low in all age groups (0.97%, 7.24% and 5.43%, respectively). In the 12-year-old adolescent group, only 3.61% had good pit and fissure sealing. In addition, the rate of dental fluorosis was 24.80%, and the Community Fluorosis Index value was 0.39. In the elder group, the community periodontal index was 2.92. The prevalence in the elderly of having lost at least one tooth was 75.54%. Additionally, 4.44% of these participants were edentulous. The incidence of dental prosthesis was 51.75%, the proportion with a removable partial denture, a fixed denture, full dentures, dental implants and an informal fixed bridge was 21.59%, 11.45%, 4.64%, 0 and 16.67%, respectively. In this study, 8.2% of the elderly participants were affected by different types of oral mucosal lesions. Among such lesions, recurrent aphthous ulcers were most prevalent (2.69%) and oral lichen planuses were second (1.6%). The conclusion presented in this survey is that oral diseases, especially dental caries and periodontal disease, are frequent and common in Sichuan province, China. Moreover, the treatment rate is very low, and primary prevention and treatment options are therefore urgently needed in this population.

## Introduction

Oral disease is one of the most prevalent chronic disease and an alarming public health problem worldwide.^1, 2^ Recently, a World Health Organization (WHO) analysis reported that oral disease has already become a determining factor for quality of life and a global burden on social and economic health.^3^ The oral health survey is the main means of obtaining oral health information that can be used to provide evidence for public health policies.^5^ Since 1971, more than 130 regions have used the methods and criteria that are recommended for inclusion in the revised Oral Health Surveys—Basic Methods. When using these inspection methods, the results are recorded in the WHO Oral Data Bank for international comparison and surveillance. Other previous national oral health surveys in China have satisfied the criteria recommended by the WHO,^4^ and the results have a high degree of lateral comparability. The methods and criteria described in the fifth edition of Oral Health Surveys—Basic Methods, which was updated in 2013, are aimed at recording the prevalence of conditions and procedures, including dental caries, periodontal status, dental fluorosis, oral mucosal disease, dentures and dental trauma.^5^

Sichuan Province, located in southwest China and inhabited by multiple ethnic groups, has a population of 81.40 million people.^6^ The economic aggregate of Sichuan ranks at first in western China and sixth in China overall. Three oral health surveys were previously conducted in Sichuan in 1983, 1995 and 2005. These surveys revealed that the prevalence of dental caries in children remained high compared with other regions, whereas in 12-year-old adolescents it was low and then gradually declined. However, most caries remain untreated and the filling rate is far from our goal. The oral status in 65–74-year-old residents is also unsatisfactory. However, rapid growth has been maintained in various clinicopathological features in Sichuan Province, including dietary structure, nutritional status and psychosocial development.^7^ Many dentists and dental clinics contribute to this progress, and awareness and the affordability of oral hygiene have improved. Moreover, the government has increased activity aimed at preventing and curing oral disease. All of these factors influence oral health status.^6^

We conducted a retrospective survey between December 2015 and May 2016, to investigate the oral health status and variations in oral health status within Sichuan Province.

## Materials and methods

The Oral Health Survey scheme was approved by the Stomatological Ethics Committee of the Chinese Stomatological Association and the Ethics Committee of West China Hospital of Stomatology, Sichuan University (Approval No. 2014-003).

### Sampling design

**Figure 1:**
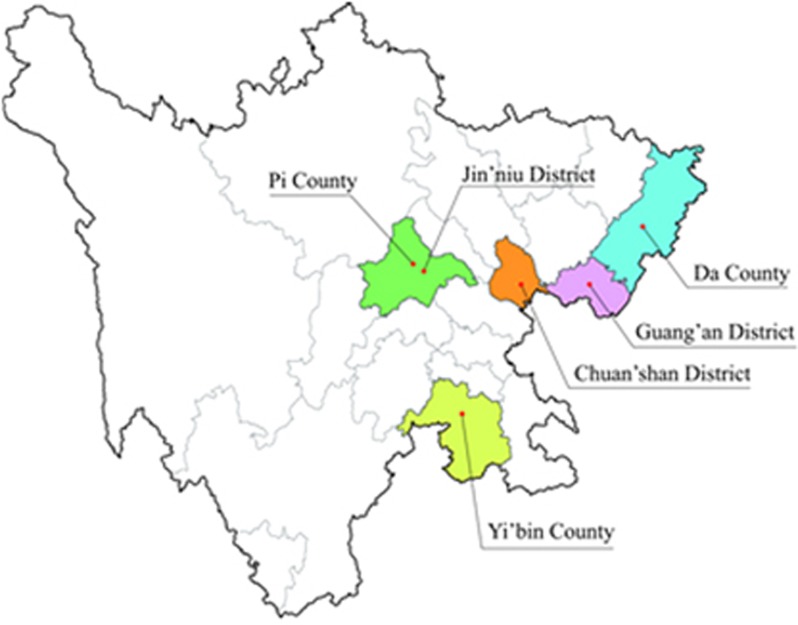
**Locations in Sichuan Province, China that were sampled in this study.**

A complex, multistage, cluster sampling design^8^ was used to select participants who were representative of the province's population. In the first stage of sampling, districts and counties were considered as strata, and population data were obtained from the 2010 census conducted by National Bureau of Statistics of the People's Republic of China. Two districts and two counties were randomly selected from each stratum using the probability-proportional-to-size (PPS)^9^ with varied population sizes method. In the second stage, because the distributions of these areas were confined to low- and middle-level urbanization, another district and county were randomly selected by PPS for good representation. Finally, six areas (Guang'an District, Chuan'shan District, Jin'niu District, Da County, Yi'bin County and Pi County) were selected for investigation in this study (Figure 1).

In the third stage, we also used the PPS method^9^ to randomly select three kindergartens and three middle schools in each area. Information such as the names and genders as well as the number of included students were obtained. This method was also used to choose three communities in each respective area. Finally, the individuals who were interviewed in the selected schools and communities were selected using a quota sampling method.^10^

### Subjects

The target population was residents aged 3–5 years, 12 years and 65–74 years in Sichuan Province. Participants must have resided in the survey area for >6 months, and ages were calculated according to the survey month.

The sample size was calculated based on the following formula:


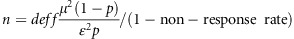


where *n* was the sample size; *deff* was the design effect (2.5); *p* was the prevalence of dental caries in each group in the Third National Oral Health Survey,^11^ which were 66.0%, 28.9% and 86.0% in 5-year-olds, 12-year-olds and 65–74-year-olds, respectively; *μ* was the level of confidence; and *ɛ* was the margin of error. The non-response rate was 20% in 3–5-year olds, 5% in 12-year olds and 15% in 65–74-year-olds. Based on this estimation, the required sample size for each age group in Sichuan Province was 2 472, 4 420 and 696, respectively. Of all, gender parity would be required.

### Clinical assessment

Every participant received a clinical assessment according to the basic methods and criteria that were issued in the World Health Organization Oral Health Survey.^5^ The contents of the clinical assessment included oral mucosa status; dental fluorosis; teeth condition, including coronal caries and root caries; denture conditions; periodontal status, including gingival bleeding, calculus; probing pocket depth and attachment loss. Children who were aged 3–5 years were assessed only for teeth condition; adolescents who were aged 12 years were assessed only for dental fluorosis, teeth condition, gingival bleeding and calculus; and participants who were aged 65–74 years were assessed for all of the described items. The examination was conducted by three trained licensed dentists and three trained individuals acted as recorders. The inspection process was performed in a mobile dental chair under artificial light using equipment including a plane mirror and Community Periodontal Index (CPI) probe.^5^

### Quality control

Before the survey, the three examiners were trained in theoretical and clinical knowledge by a standard examiner (the fourth examiner). Duplicate examinations were randomly conducted in 5% of the participants to compare the findings of examiner 1 to examiner 2, examiner 2 to examiner 3 and examiner 3 to examiner 1. Moreover, the standard examiner reviewed the data for five of the participants who were assessed by each of the other inspectors. The reviewed contents included caries, probing pocket depth of the scope for half of the teeth. If the last number of the survey ID was an odd number, we reviewed the upper right and lower left mouth, whereas for even ID numbers, we reviewed the upper left and lower right mouth. All of the review results were used to calculate a Kappa value. Before beginning the survey, each examiner enrolled three participants to calibrate the examinations. The mean Kappa values used to determine inter-examiner reproducibility were >0.80 for the periodontal exam and >0.85 for the dental caries exam.

### Statistical analysis

To reduce any data entry-related errors, the double data entry method was performed in a structured EpiData database (v3.1, EpiData Association, Odense, Denmark) in addition to validation and correction methods. All statistical analyses were performed using IBM SPSS Statistics v. 19.0 (IBM, Armonk, NY, USA). Initially, we performed descriptive analyses and group comparisons using data for characteristics, including dental caries, periodontal disease, teeth loss, pit and fissure sealing and dental fluorosis. Continuous variables are reported as the means (s.d.) or medians (range) and were compared using Student's *t*-test. Categorical variables are reported as numbers and percentages and were compared using Pearson's χ^2^ analysis.

We used the decayed, missing and filled teeth (DMFT) index to assess dental caries.^12^ This index covers the teeth and/or tooth number that were decayed, filled or extracted as a result of caries. The sum score was calculated in this study. Decayed teeth (DT), missing teeth, filled teeth (FT) and mean DMFT scores were also obtained. The percentage of participants whose DMFT=0 was used as the caries-free rate. The dental caries status is presented as the mean of dmft or DMFT. In addition, the Significant Caries Index (SiC) was calculated as the mean dmft or DMFT for the one-third of the study group with the highest caries scores.^13, 14, 15^ The caries filling constituent ratio was calculated as the restorative index using the following formula: (total number of DT/total number of DT and FT) × 100%. This index reflected the level of oral health care and the needed caries filling workload.^16^

We calculated the pit and fissure sealing rate as the ratio of subjects with sound pit and fissure sealing to all participants. The Dean Index (DI) was used to measure dental fluorosis, and a score ≥1 was the computing criteria that was used to calculate the prevalence of dental fluorosis. In this study, we calculated the Fluorosis Community Index (FCI)^17^ and the constituent ratio of different scores.

CPI was introduced by the WHO as a tool that is calculated using clinical parameters, such as sulcus bleeding index, calculus index and pocket depth.^5^ Clinical attachment loss (CAL) was determined by evaluating supporting connective tissue damage, which is a clinical pattern of periodontitis, and calculated using a CPI probe.^18^ In this study, we calculated the rate and proportion of CAL ≥4 mm in at least one recorded tooth.

## Results

### Subjects

In all, 3 000, 4 800 and 780 subjects aged 3–5 years, 12 years and 65–74 years, respectively, were selected for eligibility in this study. Table 1 presents the number of respondents who were interviewed. The non-response rate was 8.45%, 4.73% and 4.62% for participants aged 3–5, 12 and 65–74 years, respectively.

### Oral health status

#### Dental caries

Table 2 presents the prevalence rate for dental caries, the mean caries experience and the filling rate according to age and sex. The prevalence of dental caries was 63.55% in 3–5-year-olds, and the mean DMFT was 3.28 in this group. The SiC index in 3–5-year-olds reached 8.05, and this value was higher in urban areas than in rural areas. In 12-year-olds, the caries prevalence was 37.20%, the mean DMF was 0.86 and the SiC index was 2.46. This value was lower in males than in females and higher in urban areas than in rural areas. The filling rate was 7.24%, and no difference was found for either gender or residence location. In 65–74-year-olds, the dental status was used to evaluate tooth coronal and tooth root rates, with the number of decayed and filled teeth as criterion. The prevalence of dental caries was 83.20%, the mean DMFT was 5.13 and the SiC index was 11.22. The filling rate was also low in 65–74-year-olds, and only 5.42% of these participants had dental caries which were treated without secondary caries. There was an obvious difference in filling rates between rural and urban participants (0.78% *vs* 10.80%).

Table 3 shows the first permanent molar status in 12-year-old adolescents. Its caries component accounted for 70.9% of all dental caries. The prevalence of caries was 32.82%, the mean DMFT was 0.61 and the filling constitutional ratio was 8.72%. Only 3.61% of the adolescents in the 12-year-old group had sound pit and fissure sealants. This rate was higher in urban areas, where it reached 6.28%, than in rural areas.

In this study, the overall prevalence of root caries was 72.45% (67.67% in urban areas and 77.04% in rural areas, 73.48% in males and 71.47% in females, respectively). The overall mean DMFT in root caries was 4.10 (3.48 and 4.69 in urban and rural areas, respectively). The filling rate was 1.77%, and this value was higher in females in urban areas (Table 4).

#### Dental fluorosis

The incidence of dental fluorosis was 24.80% (DI≥ 1; *n*=1 134) in 12-year-olds. The FCI in this group was 0.39, and the popular status was negative with the DI. These results are presented in Table 5.

#### Periodontal status

In the 12-year-olds, periodontal status was determined only as checked gingival bleeding and calculus. In all, 46.71% (*n*=2 136) of these subjects had gum bleeding on probing. The mean teeth number in this group was 4.01. The prevalence of calculus was 66.85% (*n*=3 055) and the mean teeth number in patients with calculus was 4.56.

The CPI of elderly participants was 2.92 in the statistical analysis. In this study, 80.78% (*n*=598) of the elderly participants had a clinical pattern of attachment loss (defined as 4 mm). The results above presented in Table 6, 7.

Not including the third molar, 75.54% of the elderly had at least one tooth missing, and the edentulous rate in this group was 4.44%. The average number of teeth lost was 5.81 (4.66 in urban areas and 6.92 in rural areas, 5.52 in males and 6.08 in females, respectively). The denture repair rate was 51.75%, and this value was higher in urban areas than in rural areas. In the elderly population, the prevalence of removable partial dentures, fixed dentures and full dentures were 21.59% (*n*=161), 11.45% (*n*=85) and 4.64% (*n*=34), respectively. Additionally, 16.67% (*n*=123) of these participants had received an informal fixed bridge. No dental implants were observed in this study population. These results are shown in Table 8.

The incidence of oral mucosal disease was 8.2% (*n*=61). Recurrent aphthous ulcers were most common in these patients (prevalence, 2.69% *n*=20). Oral lichen planus was the second most common condition of all oral mucosal diseases (prevalence, 1.6% *n*=12). The constituent ratios of oral disorders are presented in Table 9.

## Discussion

The mean DMFT in 12-year-olds was a significant indicator of oral health status.^19^ This statistic was determined by dividing the caries level into four grades. The mean DMFT observed in this study (0.86) was lower than that reported in previous studies that have included data obtained in the western Pacific region^20, 21^ (http://www.mah.se/CAPP/Country-Oral-Health-Profiles/According-to-Alphabetical/Global-DMFT-for-12-year-olds-2011/). The WHO has established Oral Health Goals for the year 2020. While these goals include a no-caries rate <50% in individuals aged 5 years,^22^ the rate observed in this study was 63.47%, and the mean DMFT was 3.28. The prevalence of caries in 3- and 4-year-olds was already >50%, and this phenomenon reveals that there is a high level of dental caries in children in this region. The mean CPI score was 2.92, and approximately 75.43% (*n*=558) of the included individuals exhibited a pocket depth of >4 mm. Compared with the latest WHO Oral Data Bank data for Japan and Mongolia,^23^ the periodontal status of residents in Sichuan Province is more severe, and these individuals have a higher rate of periodontal pockets. The results of this investigation reveal that dental decay and periodontal disease are the most prevalent and alarming problems that are currently affecting oral health in Sichuan Province. Moreover, other oral diseases, such as oral mucosal disease and dental fluorosis, are common in these residents, and the facts revealed in this study demonstrate that oral health should receive more attention in this population.

When we analyzed the data obtained in the three oral health surveys conducted in Sichuan Province, differences in oral health status were clear.^24^ In 5-year-old children, the caries prevalence increased from 66.0% to 71.9%, and the mean dmft rose from 2.71 to 4.23. The prevalence of caries in 12-year-old adolescents changed from 28.5% to 37.2%, and the mean DMFT changed from 1.02 to 0.86. The caries level in 65–74-year-olds also increased, with the caries prevalence rising to 83.2% and mean DMFT rising to 5.13. Moreover, the prevalence of periodontal packets was higher than that reported in previous studies, and the edentulous rate fell from 6% to 4.44% (Table 10). A growing number of natural teeth was one benefit that has resulted from improvements in health care and oral health awareness. Although the factors and pathogenesis underlying these changes require further study, their identification will lead to more effective treatment and prevention options.

There is a clear difference in oral disease prevention and treatment between urban and rural areas. The filling rates in 3–5-year-olds were 1.50% and 0.49% in urban and rural areas, respectively, and in 12-year-olds, the pit and fissure sealing rates were 6.28% and 0.96%, respectively. The filling rate in 65–74-year-olds was 10.8% in urban areas and nearly 0 in rural areas. Moreover, the denture repair rates were 58.63% and 45.11% in urban and rural areas, respectively. These differences may be associated with differences in the level of economic development, the distribution of oral health human resources and oral health awareness within each areas.^25, 26^

The filling rate was very low in 3–5-, 12- and 65–74-year-old groups (0.98%, 8.00% and 5.42%, respectively), and low index scores indicated that many teeth with caries had not been effectively treated. The CPI index in the elderly was 2.92, and CPI3 and CPI4 accounted for 74.53% of these cases (*n*=552). CPI scores reflect not only a bad oral health status but also a strong need for treatment. About three-fourths of the subjects in the 65–74-year-old group had a tooth missing. In half of these patients, the teeth were not repaired; the need for dentures in the elderly is therefore very high. Over one-fourth of elderly denture wearers had an informal fixed bridge, and urgent treatment is needed in these patients as well. Similar to the rest of the world, oral health status is not good and the associated burden of medical care is high in Sichuan Province. In summary, the high need for treatment and the heavy burden this places on many human and medical resources continues to make it hard to reach oral health goals in China.

In 12-year-olds, first permanet molars are at high risk of having caries, and the data in this study reveal that permanent teeth caries account for 71.2% of all dental caries. There is first evidence showing that sealants are effective in preventing pit and fissure caries, but only 3.61% of adolescents in the 12-year-old group had sound pit and fissure sealing. The promotion and wider practice of this procedure is therefore urgently needed.^23^ Poor oral health status was observed in all age groups, with 46.7% and 66.8% of the 12-year-old adolescents exhibiting gum bleeding on probing and calculus, respectively. These numbers were 78.8% and 91.7%, respectively, in the 65–74-year-old group. These phenomenon should be important to dental professionals and policy makers, who should focus on strengthening primary prevention in the public. Early diagnosis of caries and the early performance of risk assessments to identify caries-active individuals have been shown to be the most effective interventions for preventing future dental procedures.^27^ The application of pit and fissure sealants and topical fluoride, which are wildly used to prevent decay,^28^ should have a vital role in improving the caries status of individuals residing in Sichuan Province. Periodontal status could also be improved by implementing initial and other necessary periodontal therapies.^29, 30^

In conclusion, oral health status is not ideal and dental caries, periodontal disease, dental fluorosis and oral mucosal disease are common characteristics of various age groups in Sichuan, China. Therefore, the development of in-depth primary health care is urgently needed in this population.^27^ Analyses of the risk factors that contribute to oral disease could promote the development of preventive and treatment strategies. The national oral health survey is conducted each decade in China, and this could be used to efficiently supervise and track improvements in oral health status and its variations.

## Figures and Tables

**Table 1:** Demographic characteristics of subjects included in the oral health survey in Sichuan Province

	Gender		Residence location	
Age/years	Male	Female	Urban	Rural
3–5 (*n*=2 746)	1 384	1 362	1 425	1 321
12 (*n*=4 573)	2 227	2 346	2 278	2 295
65–74 (*n*=744)	362	382	365	379

**Table 2:** Dental status in Sichuan Province in 2016

	3–5 years				12 years				65–74 years			
Items	Prevalence of dental caries/%	Mean dmft	SiC	Filling ratio/%	Prevalence of dental caries/%	Mean DMFT&dmft	SiC	Filling ratio/%	Prevalence of dental caries/%	Mean DMFT	SiC	Filling ratio/%
Total	63.47	3.28	8.05	0.97	37.20	0.86	2.46	7.24	83.20	5.13	11.22	5.43
Gender												
Male	62.79	3.33	8.24	0.59	30.89	0.64	1.92	6.80	82.60	4.95	11.07	4.08
Female	64.17	3.22	7.86	1.37	43.18	1.07	2.90	7.48	83.77	5.30	11.33	6.62
Residence location												
Urban	61.54	3.00	7.46	1.50	42.27	1.05	2.88	6.19	82.74	4.85	10.73	10.80
Rural	65.56	3.58	8.59	0.49	32.16	0.67	2.00	8.86	83.64	5.40	11.66	0.78

DMFT, decayed, missing and filled teeth; SiC, Significant Caries Index.

**Table 3:** First permanent molar status in 12-year-old adolescents

Items	Prevalence of dental caries/%	Mean DMFT&dmft	The filling rate/%	Pit and fissure sealing rate/%
Total	32.82	0.61	8.72	3.61
Gender				
Male	27.21	0.47	7.54	3.41
Female	38.15	0.75	10.57	3.79
				
Residence location				
Urban	37.40	0.75	7.42	6.28
Rural	28.28	0.47	9.49	0.97

DMFT, decayed, missing and filled teeth.

**Table 4:** Root caries status in 65–74-year-olds

Items	Prevalence of dental caries/%	Mean DMFT	The filling rate/%
Total	72.45	4.10	1.77
Gender			
Male	73.48	4.13	0.94
Female	71.47	4.06	2.58
			
Residence location			
Urban	67.67	3.48	4.26
Rural	77.04	4.69	0.00

DMFT, decayed, missing and filled teeth.

**Table 5:** Dental fluorosis status in 12-year-olds

			Constituent ratio/%					
Items	Prevalence/%	CFI	DI=0	DI=0.5	DI=1	DI=2	DI=3	DI=4
Total	24.80	0.39	64.08	11.08	17.89	5.83	0.83	0.28
Gender								
Male	24.56	0.39	64.40	11.01	17.39	6.16	0.72	0.31
Female	25.02	0.39	63.78	11.15	18.37	5.51	0.94	0.26
								
Residence location								
Urban	16.94	0.27	74.90	8.13	11.69	4.79	0.40	0.09
Rural	32.59	0.50	53.34	14.01	24.05	6.85	1.27	0.48

CFI, Community Fluorosis Index; DI, Dean Index.

**Table 6:** Periodontal status in 65–74-year-olds

		Prevalence of persons with highest score						
Items	CPI	0	1	2	3	4	Mean number of teeth with CAL	Prevalence of persons who have CAL
Total	2.92	0.29	0.29	24.89	55.90	18.63	7.05	80.78
Gender								
Male	2.98	0.29	0.58	20.47	57.89	20.76	7.93	85.63
Female	2.86	0.29	0.00	29.28	53.91	16.52	6.20	76.18
								
Residence location								
Urban	2.88	0.29	0.29	30.09	50.14	19.20	7.16	81.37
Rural	2.97	0.30	0.30	19.53	61.83	18.05	6.93	80.21

CAL, clinical attachment loss; CPI, Community Periodontal Index; PD, pocket depth; 0, no disease; 1, bleeding on probing; 2, calculus; 3, PD 4–5 mm; 4, PD≥6 mm.

**Table 7:** Periodontal status in 12-year-olds

	Gum bleeding on probing		Calculus	
Items	*N*	Prevalence/%	*N*	Prevalence/%
Total	4.01	46.71	4.56	66.85
Gender				
Male	4.02	46.97	4.81	70.09
Female	4.00	46.46	4.33	63.77
Residence location				
Urban	2.65	40.21	3.85	64.40
Rural	5.36	53.16	5.27	69.28

**Table 8:** Missing teeth and denture repair rates in 65–74-year-olds (not including third molars)

Items	Number of missing teeth	Missing tooth rate/%	Prevalence of endontulism/%	Denture repair rate/%	Prevalence of informal fixed restorations/%
Total	5.81	75.54	4.44	51.75	16.67
Gender					
Male	5.52	75.97	2.49	48.90	16.02
Female	6.08	75.13	6.28	54.45	17.28
					
Residence location					
Urban	4.66	71.78	2.74	58.63	17.68
Rural	6.92	79.16	6.07	45.11	15.62

**Table 9:** Constituent ratios of oral disorders

Items	OLK	OLP	RAU	Candidiasis	Abscess or fistula	Hemangioma	Keratosis	Mucinous cyst	Pigmented nevus	FT
Detection rate/%	0.67	1.61	2.69	0.40	0.81	0.54	0.54	0.40	0.13	0.13

FT, fissured tongue; OLK, oral leukoplakia; OLP, oral lichen planus; RAU, recurrent aphthous ulcers.

**Table 10:** Dental caries status in 1995, 2005 and 2015

	Caries prevalence/%			Mean DMFT & dmft		
Age/years	1995	2005	2015	1995	2005	2015
5	66.0	58.7	71.9	2.71	2.77	3.83
12	28.5	25.7	37.2	1.02	0.50	0.86
65–74	61.5	81.4	83.2	2.81	5.04	5.13

DMFT, decayed, missing and filled teeth.
